# EXAMINE QUALITY OF CARE AMONG OLDER ADULTS IN MEDICARE ADVANTAGE COMPARED WITH TRADITIONAL MEDICARE

**DOI:** 10.1093/geroni/igae098.4310

**Published:** 2024-12-31

**Authors:** Elham Mahmoudi

**Affiliations:** University of Michigan Medical School, Ann Arbor, Michigan, United States

## Abstract

About 50% of Medicare beneficiaries are enrolled in Medicare Advantage (MA), incentivized to offer more efficient and coordinated care than traditional Medicare (TM). Evidence suggests timely access to coordinated and high-quality ambulatory care is critical to preventing adverse health events (e.g., hospitalization and long-term institutionalization). In this study, we used 2017-2021 (latest available) Medicare Current Beneficiary Survey (MCBS) data to examine the effect of MA vs. TM on 10 quality metrics among older adults with and without dementia. We applied inverse propensity weighting to balance the characteristics of MA and TM enrollees and used the Average Treatment Effect method, adjusting for sociodemographic, rurality, and health-related variables. Among people without dementia, MA was associated with greater challenges receiving needed care [TM=5.06%; MA=6.50%; (diff=1.4 percentage point (PP); 95% CI:0.9%,1.9%)] and accessing specialty care [TM=5.25%; MA=6.02%; (diff=.73 PP; 95% CI:.21%,.1.26%)]. However, TM was associated with more dissatisfaction with out-of-pocket costs [TM: 13.78%; MA=12.18%; (diff=1.6PP; CI: -2.32%, -.87%)] and distance/location of different health services [TM=8.67%; MA=7.49%; (diff=1.18 pp; CI: -1.81, -.55)]. Among beneficiaries with dementia, except for dissatisfaction with ease of access to care with better results in MA (TM=7.53%; MA=5.09%; diff=2.45pp; 95%CI: -4.6%, -.26%), we did not find any significant differences in quality metrics between MA and TM. In conclusion, TM performed better in receiving the needed care and specialty care. MA was superior in lowering out-of-pocket costs and better care coordination. Dissatisfaction with care was substantially higher among enrollees with dementia than among those without, regardless of MA or TM.

